# Implications for health system reform, workforce recovery and rebuilding in the context of the Great Recession and COVID-19: a case study of workforce trends in Ireland 2008–2021

**DOI:** 10.1186/s12960-022-00747-8

**Published:** 2022-05-26

**Authors:** Padraic Fleming, Steve Thomas, Des Williams, Jack Kennedy, Sara Burke

**Affiliations:** 1 Centre for Health Policy and Management, Trinity College Dublin, The University of Dublin, 3-4 Foster Place, Dublin 2, Ireland; 2 National Human Resources Directorate, HSE, Dr. Steevens’ Hospital, Dublin 8, Ireland

**Keywords:** Health system shock, Financial crisis, COVID-19, Resilience, Workforce, Reform, Universal healthcare delivery

## Abstract

**Background:**

Workforce is a fundamental health systems building block, with unprecedented measures taken to meet extra demand and facilitate surge capacity during the COVID-19 pandemic, following a prolonged period of austerity. This case study examines trends in Ireland’s publicly funded health service workforce, from the global financial crisis, through the Recovery period and into the COVID-19 pandemic, to understand resource allocation across community and acute settings. Specifically, this paper aims to uncover whether skill-mix and staff capacity are aligned with policy intent and the broader reform agenda to achieve universal access to integrated healthcare, in part, by shifting free care into primary and community settings.

**Methods:**

Secondary analysis of anonymised aggregated national human resources data was conducted over a period of almost 14 years, from December 31st 2008 to August 31st 2021. Comparative analysis was conducted, by professional cadre, across three keys periods: ‘Recession period’ December 31st 2008–December 31st 2014; ‘Recovery period’ December 31st 2014–December 31st 2019; and the ‘COVID-19 period’ December 31st 2019–August 31st 2021.

**Results:**

During the Recession period there was an overall decrease of 8.1% (*n* = 9333) between December 31st 2008 and December 31st 2014, while the Recovery period saw the overall staff levels rebound and increase by 15.2% (*n* = 16,789) between December 31st 2014 and December 31st 2019. These figures continued to grow, at an accelerated rate during the most recent COVID-19 period, increasing by a further 8.9% (*n* = 10,716) in under 2 years. However, a notable shift occurred in 2013, when the number of staff in acute services surpassed those employed in community services (*n* = 50,038 and 49,857, respectively). This gap accelerated during the Recovery and COVID-19 phase. By August 2021, there were 13,645 more whole-time equivalents in acute settings compared to community, a complete reverse of the 2008 situation. This was consistent across all cadres. Workforce absence trends indicate short-term spikes resulting from shocks while COVID-19 redeployment disproportionately impacted negatively on primary care and community services.

**Conclusions:**

This paper clearly demonstrates the prioritisation of staff recruitment within acute services—increasing needed capacity, without the same commitment to support government policy to shift care into primary and community settings. Concerted action including the permanent redistribution of personnel is required to ensure progressive and sustainable responses are learned from recent shocks.

## Background

The health workforce is a fundamental building block within health systems, and that has never been more evident than during the COVID-19 pandemic when the workforce tackled extraordinary demand with undermined supply [1, 2]. Health services across the globe have been taking unprecedented measures to meet extra demand and facilitate surge capacity, such as widespread recruitment of medical students, retired health professionals and volunteers [3]. Simultaneously, workforce supply has been hindered by increased absence-rates, directly linked to COVID-19 infection, but also due to burnout after sustained high-pressure work environments—requiring proactive measures to protect staff’s physical and mental health [4].

The COVID-19 pandemic arrived as health systems emerged from the austerity era that followed the 2008 financial crisis. The global recession had a detrimental impact on many European health systems, in terms of lower financial investments in healthcare, and specifically on the health workforce, with increased stress and burn-out regularly reported [5–7]. Health workforce resilience was pushed to its limit, not least due to staff shortages resulting from restrictive policies and budgets [8, 9]. For example, the recruitment moratorium introduced in Ireland in March 2009 [10] set an expectation that public health systems and staff could consistently ‘do more with less’ [11].

The adverse impact of prolonged budget constraints also impacted healthcare delivery more broadly, with evidence that community-based health care delivery was compromised. Many countries, for example, experienced increased use of hospital emergency departments, largely due to free emergency care compared to out-of-pocket payments associated with community-based primary care [12–15]. There were early attempts to counteract this trend. In Portugal for example, primary care utilisation was incentivised by lowering co-payments associated with primary care [12]. Nevertheless, this shift towards acute settings created challenges for countries with health systems modelled on universal healthcare delivery, although evidence suggests that universal healthcare systems were more resilient during austerity, particularly in terms of equitable access [16–18]. Despite challenges imposed by the prolonged period of austerity in Ireland, a radical policy shift towards universal healthcare was underway when the COVID-19 pandemic began, with a policy-driven plan for community-based delivery of care. This ambitious health reform programme, known as Sláintecare, is firmly grounded in international best practice, with Ireland playing catch up with most European countries, particularly in terms of delivering universal access to integrated healthcare [19]. In addition, Sláintecare aims to address inequitable and poor healthcare access, and to shift care into primary and community settings through population-based resource allocation; planning and delivering integrated care within regions; free general practitioner (GP), primary and in-patient care; a reduction in waiting times to access diagnosis and care; the removal of private care from public hospitals; and considerable expansion of public health initiatives [20].

Two years before the COVID-19 pandemic, a Health Service Capacity Review was undertaken [21], followed by several key policy documents, service delivery plans and implementation strategies, in both the Health Service Executive (HSE) and Department of Health (DoH). These policy documents consistently highlighted the need to shift care from hospitals to community-based settings to bring care closer to home, with sufficient workforce capacity and skill-mix to see that these changes made possible [21–25]. Indeed, policy intent to move towards integrated community care can be tracked back several decades in Ireland [26]. While there is cross-party political support for Sláintecare, progress has been slow, although the recent COVID-19 pandemic reportedly saw the government utilise the opportunity to fast-track changes largely aligned with the reform programme. A recent policy analysis highlighted some of these initiatives to advance the reform agenda, such as an influx of funding to provide free, and universal access to COVID-care; the introduction of new hospital beds; new consultant contracts; and the long-awaited introduction of ‘Individual Health Identifiers’ via the COVID-19 vaccination programme, in order to advance digital interoperability within the health sector [20].

The analysis presented in this article was conducted during the early implementation of Sláintecare, spanning two separate but interconnected research programmes. The first was examining the impact of COVID-19 on health reform in Ireland (2019–2022) [27], while the second took a broader view of health system resilience when faced with successive shocks, including the impact on reform (2020–2025) [28]. Given the aforementioned contextual factors impacting health service resourcing, specifically human resourcing, this paper takes Ireland’s publicly funded health service workforce as a case study. Health service staffing trends were examined from the beginning of the global financial crisis, through the Recovery period that followed and into the COVID-19 pandemic (to August 2021), with a view to understanding resource allocation, particularly in terms of professional cadres across community and acute settings. Specifically, this paper aims to uncover whether skill-mix and staff capacity are aligned with policy intent and the broader reform agenda.

## Methods

Secondary analysis of anonymised aggregated national human resources data was conducted over a 14-year period, from 2008 to August 2021. Following an earlier collaboration and analysis between the Centre for Health Policy and Management and the HSE [10], staffing data, pertaining to the public health system, were made available by the national HSE Human Resources division in Ireland. The workforce is defined as directly employed whole-time equivalent (WTE) public service staff in the HSE and other agencies encompassed by section 38 of the Heath Act (2004), as covered by Department of Public Expenditure and Reform public service employment numbers and the Government Employment Control Frameworks [10].

Comparative analysis was conducted across three keys periods, namely the ‘Recession period’ December 31st 2008–December 31st 2014; the ‘Recovery period’ December 31st 2014–December 31st 2019; and the ‘COVID-19 period’ December 31st 2019–August 31st 2021. Individual roles were grouped into six overarching cadres as categorised by HSE Human Resources division: (1) Medical and Dental: consultants, registrars, senior house officers, interns, and dentists; (2) Nursing and Midwifery: staff nurses/midwives, managers, specialists, advanced nurse practitioners, public health nurses, and students (post-registration/pre-registration clinical); (3) Health and Social Care Professionals: therapy professionals, health science/diagnostics, social care, pharmacy, psychologists, and social workers; (4) Management and Administration: all clerical, administrative and management grades; (5) General Support: catering, household, porters, maintenance, and technical services; and (6) Patient and Client Care: health care assistants, home help, and ambulance staff. While there are recognised limitations related to aggregated analysis of health workforce data, making it difficult, for example, to distinguish between frontline and support staff or to conduct comparative analysis between countries since categorisation may differ [29]. However, this approach is aligned with national reporting of HR data, as defined by the national HSE Human Resources division and previous analysis in Ireland [10]. Furthermore, the current categorisation was also organised by acute and community setting, allowing for the necessary comparative analyses. Apart from 2021, yearly datasets were captured on December 31st when the best comparable data were available, with seasonal fluctuations related to temporary staff (interns, students) accounted for. Finally, annual absence-rates were based on the average monthly absence-rate within a given year, representing the best available data.

Data were collated and cleaned by the HSE Human Resources division (data controllers), while comparative trend analysis was independently conducted by researchers in Trinity College Dublin, utilising Microsoft Power BI and Microsoft Excel for analysis and data visualisation. Given the nuanced nature of the dataset, all interpretations were sense-checked with the data controllers to ensure accuracy, as well as with knowledge users within the Sláintecare reform programme in the DoH and the HSE.

### Data constraints

On January 1st 2014, the Child and Family Division of the DoH and Children transferred its 3,390 WTE staff (3.2% of HSE staff at the time) to the Child and Family Agency. This reduction of total WTE slightly skews direct comparisons between the Recession period and subsequent periods, however it does not affect the overall trends observed. These staff are still included in 2008–2014 (inclusive) figures presented here, from 2015 onwards the figures only include HSE staff and not staff associated with Child and Family Services. An adjustment has been made to 2014 data for the Recovery period to ensure year-on-year comparisons are accurate. Where a specific year is accompanied by asterisk (*), this indicates that the reported figure includes the 3390 staff, for comparative purposes. Finally, given the analysis is based on WTE rather than headcount, it was not possible to observe temporary changes due to, for example, part-time staff temporarily moving to full time although related readjustments may be reflected in future analysis, post-pandemic.

## Results

### Overall staffing levels

During the Recession period (December 31st 2008–December 31st 2014) there was an overall decrease of 8.1% (*n* = 9333) between 2008* and 2014*, while the Recovery period (December 31st 2014–December 31st 2019) saw overall staff levels rebound and increase by 15.2% (*n* = 16,789). These figures continued to grow, at an accelerated rate during the most recent COVID-19 period (December 31st 2019–August 31st 2021), increasing by a further 8.9% (*n* = 10,716) in under 2 years. The overall increase from 2014 to August 2021 was 24.9% (*n* = 27,505) although the increase is less marked when compared to pre-recession figures (increasing by 12.8% between 2008* and August 2021).

**Fig. 1:**
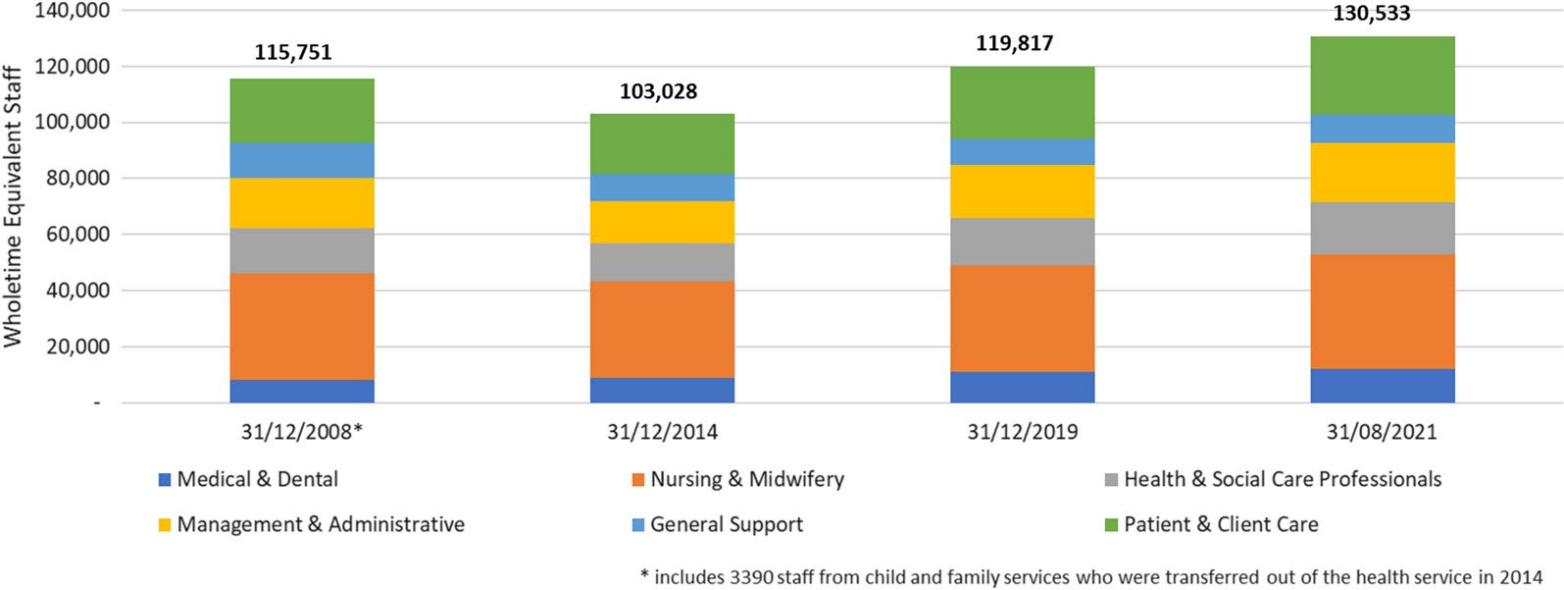
Distribution by staff category as proportion of overall WTE

We can see that across the six major staff categories the distribution of staff, as a proportion of overall WTE, remained consistent across most categories (Fig. 1), with the number of WTE personnel rapidly increasing by 25% over the 7-year period (2014–2021).

### Changes by staff group

**Fig. 2:**
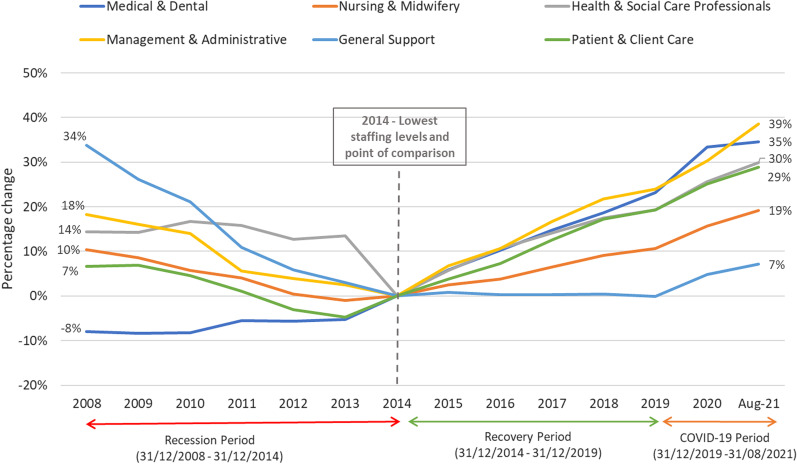
Percentage change within staff category pre- and post-2014

The lowest staff numbers were recorded in 2014 (*n* = 103,028) and coincides with the end of the Recession period and beginning of the Recovery period. 2014 is also used as a point of comparison in Fig. 2. Examining changes in WTE within each major staff category across the three periods (Fig. 2), a general downward trend can be seen across most staff categories during the Recession period, apart from Medical and Dental, which increased by 8% between 2008 and 2014 (*n* = 708). This can be partly explained by the exemption of certain frontline staff from the recruitment freeze implemented as a control instrument to reduce public sector staff, including medical consultants, therapists (physio, occupational and speech-language) and social workers [10]. As a result, Health and Social Care Professionals also remained stable (Fig. 2), actually increasing earlier in the Recession period (2009–2010), before a sudden drop of 14% (*n* = 2204) between 2013 and 2014, with the transfer of staff to the Child and Family Division of the DoH and Children. Among all staff categories, General Support (porters, attendants, catering, and cleaning) experienced the greatest decrease (34%) over the Recession period (*n* = 3198).

Moving to the Recovery period, from 2014 to 2019 (Fig. 2), a steady increase was seen across most groups, except for ‘General Support’, which remained largely static until 2019 (*n* = 9419 and 9416, respectively), partly explained by task-shifting and outsourcing, before it increased sharply by 7.1% (*n* = 674) by August 2021. This sharp increase was repeated across all categories, likely to be a direct response to the COVID-19 pandemic, although the number of Medical and Dental staff appears to have plateaued in 2020.

### Acute/community split

**Fig. 3:**
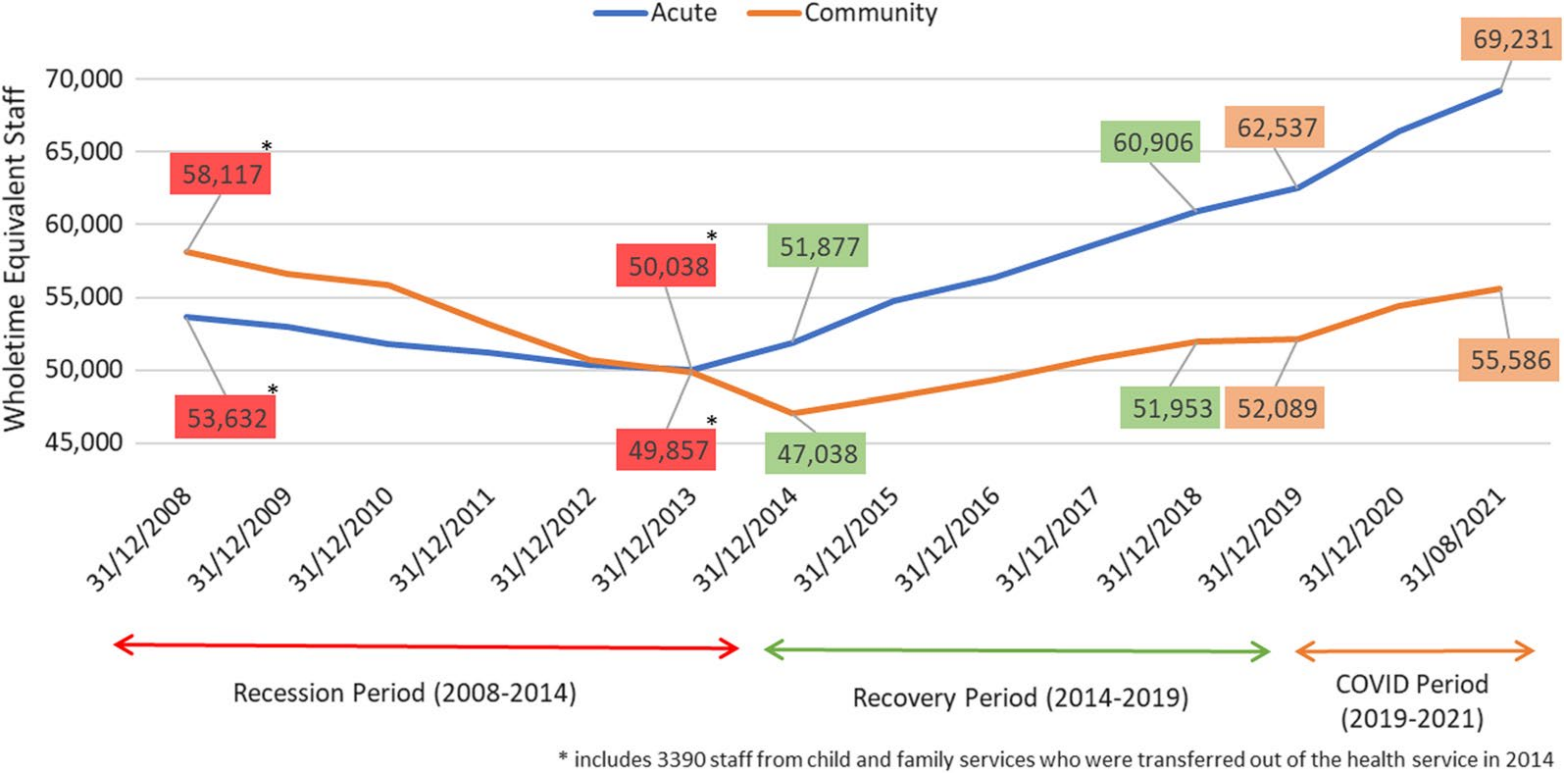
Trends in acute and community staffing levels 2008—August 2021

Before the financial crisis, there were considerably more WTE staff based in community services compared to acute settings (a difference of 4485, 4%). While all staff numbers dropped during the Recession period, the decline was sharper in community-based services (Fig. 3). A notable shift occurred in 2013, when the number of staff in acute services surpassed those employed in community services (*n* = 50,038 and 49,857, respectively). Exacerbated by the transfer of staff to the Child and Family Division of the DoH and Children in 2014, the gap continued to widen throughout the Recovery period. From 2014 to 2019, the greatest staff gains were within acute settings, increasing by over a third from the beginning of the Recovery period (*n* = 17,354, 33.5%), compared to an 18.2% increase for community services over the same period (*n* = 8548). This gap accelerated during the COVID-19 period. By August 2021, the gap had tripled with 13,645 more WTEs in acute settings compared to community, a complete reverse of the 2008 situation, when the numbers were weighted in favour of community settings (Fig. 3).

**Fig. 4:**
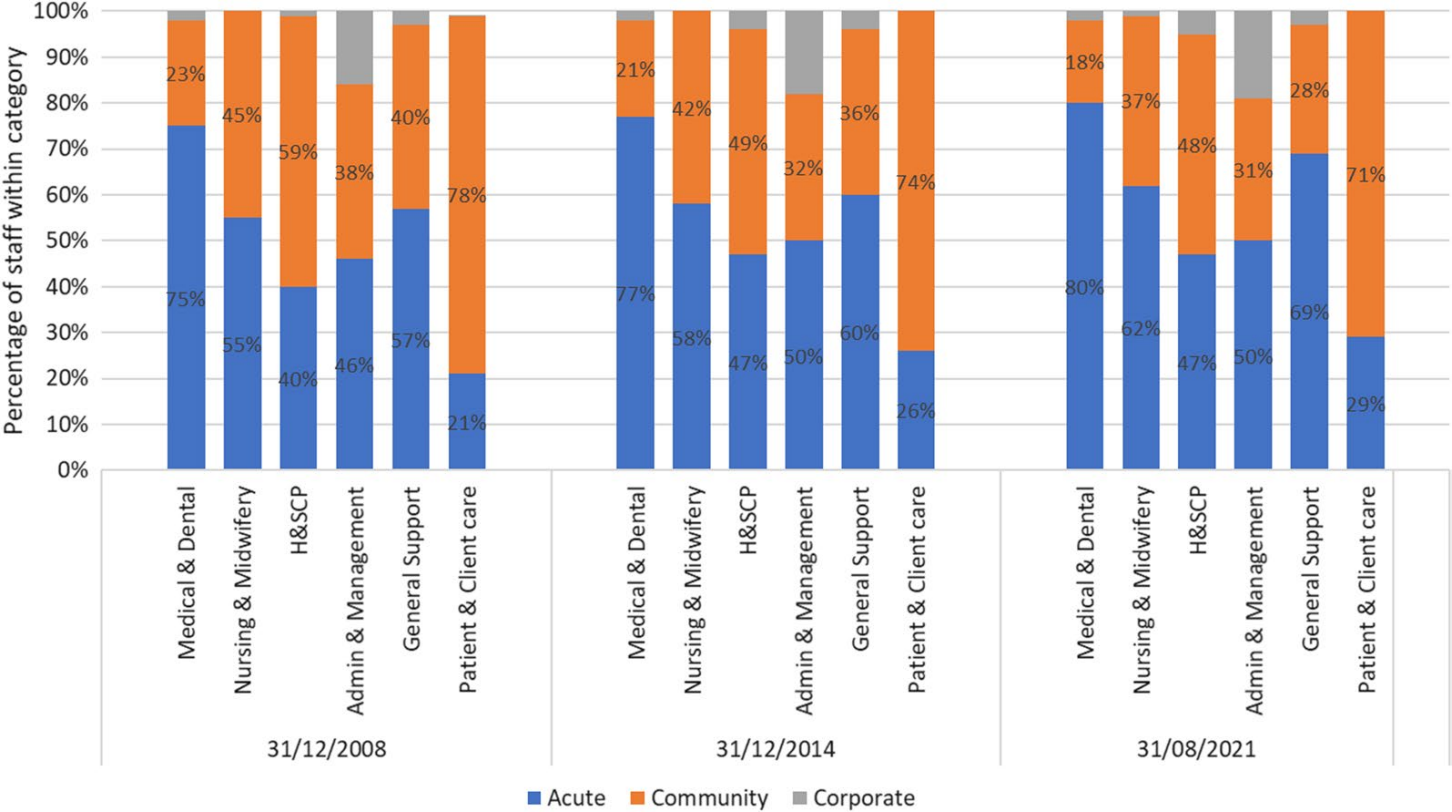
Staff categories distributed across acute, community and corporate settings

The overall trend towards increased staffing of acute services was investigated further by examining the proportion of staff in acute, community and corporate services across the six major staff categories (Fig. 4). These data are presented for 2008, 2014, and 2021 showing a clear shift towards staffing acute services across all six categories over time. General Support staff saw the greatest decrease (12%) in community services between 2008 and 2021, followed by Health and Social Care professionals (11%). Nursing and Midwifery saw WTE staff in community settings decrease by 8%, with Patient and Client Care, and Administration and Management both decreasing within community settings by 7% each. Unsurprisingly perhaps, Medical and Dental staff saw the smallest change over the study period, with a 5% reduction in community services. Staff reductions in community services represent staff gains for acute settings, as demonstrated in Fig. 4.

### Absence-rates

While examining WTE is useful for understanding macro-level resource allocation and prioritisation, ultimately, it is important to understand the level of staff who are consistently at work, providing care during ‘normal’ times and during a health system shock. Tracking and understanding absence is therefore useful for determining shorter term resource allocation pressures during periods of intense upheaval, which can ultimately influence longer term decisions regarding the balance between appropriate skill-mix and capacity during ‘normal’ times, compared to having sufficient surge capacity in preparation for shock onset. By analysing staff absence-rates over the three study periods, it is clear that absence is directly impacted by shocks, albeit with potentially different causes.

**Fig. 5:**
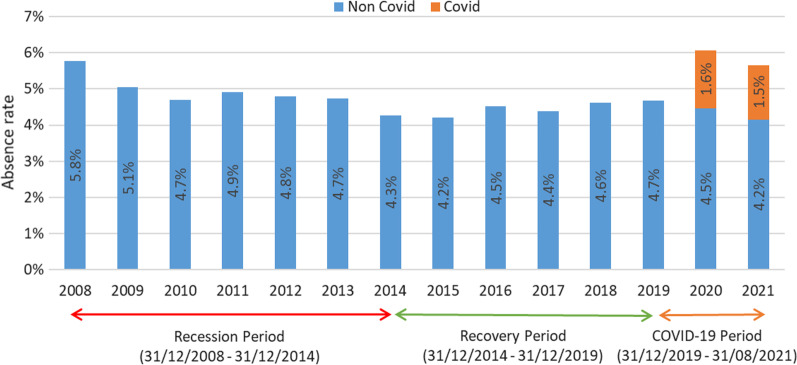
Staff absence-rates 2008—August 2021

Absence reached a high at the beginning of the financial crisis in 2008 (5.8%) and dropped to a low of 4.2% during the Recovery period, while increasing substantially to 6.1% during the COVID-19 period. Notably, while overall absence-rates peaked during COVID-19, non-COVID-19 absences dropped below average (Fig. 5). More granular data from the COVID-19 period show that absence reached a high of 10.4% in March 2020, in the very early stages of the pandemic, up 6% from the previous year (4.4% in March 2019), with the highest absence-rate by staff category seen in Nursing and Midwifery (8.2%), followed by Patient and Client Care (6.6%) [30]. While absence began to stabilise in the latter half of 2020, another spike of 9.3% was seen in January 2021 [30], in line with the third and most serious wave of the pandemic in Ireland—during the timeframe under consideration, when Ireland recorded the highest global weekly rate of infection per 1000 population during the second week in January 2021 [20].

### Redeployment during COVID-19

Another short-term response to a shock is to redeploy staff [31]. The national dataset is based on contract of employment, including geographical location of an individual’s place of employment, thereby limiting its scope in terms of observing temporary redeployment changes during COVID-19. While very little data pertaining to staffing issues specific to the COVID-19 period are currently available, the National Public Health Emergency team published redeployment data during the initial months of the COVID-19 pandemic (April–November 2020). During this time, a large number of community staff were redeployed to COVID-19 response services (serving both acute and community needs) while recruitment of dedicated contact tracers and swabbers was underway [32]. In April 2020, 3555 WTE community staff were redeployed (7.2% of all WTE community staff), and this was eventually reduced to 815 (1.7%) by November 2020. This redeployment, at least in the short term, was recognised as placing a strain on non-COVID-19 primary care and community services, ultimately reducing capacity to deliver services, having “challenged the resilience of the primary care workforce as a whole” [33].

## Discussion

### Policy context

Despite consistent policy recommendations to shift care from hospitals to community-based settings [24, 25], the findings from this study clearly demonstrate an ever-growing gap between the number of community-based WTE staff, compared to those in acute settings. Trends were undoubtedly shaped by the two major global shocks occurring during the study period. The 2008 financial crisis had a debilitating effect on workforce numbers, bringing many health systems under extreme pressure through restrictive programmes of austerity. In Ireland, governance control measures to introduce efficiencies (for example the recruitment moratorium) protected certain (mainly clinical) roles by making them exempt from restrictive policies, pointing to a systemic bias towards acute care that continues to present day. The second, the COVID-19 pandemic, saw a huge investment in staff, albeit in areas driven by the crisis response rather than policy.

While the shocks were undoubtedly very different, responded to by fiscal and then public health measures, their impact on the current reform agenda reveals a conflict between policy intent of transferring more care to the community and implementation on the ground. Unlike the Recession period, the COVID-19 response was anything but austere, with strategies adopted across Europe and the world to build, retain and strengthen the health workforce [3], made possible by huge financial investments [34]. Key trends included rapid recruitment drives, redeployments, retraining and retention strategies put in place to ensure health services were well equipped to deal with a surge in demand, while also ensuring capacity to protect staff health and well-being [35]. Again, these supportive policies were far removed from recession-driven policies, when a culture of ‘presenteeism’ was promoted, and where staff often attended work when unwell, which led to a deterioration of working conditions, frustration, burn-out, and ultimately emigration in some cases [36]. In contrast, illness-cover was arranged during COVID-19 to allow staff to take sick leave or isolate, when necessary [37].

In line with the more supportive initiatives for existing staff during COVID-19, the overall size of the health workforce in Ireland was bolstered during the pandemic. These surge capacity measures, however, resulted in an accelerated divergence between acute and community settings, the gap having tripled in size compared to that seen in 2008. In fact, despite the recent COVID-19 spike in recruitment, against a policy backdrop that emphasises delivery of care outside of hospitals, official staff numbers in community settings have still not recovered to pre-recession levels. This surge in acute staff is in direct opposition to commitments made in terms of community-based staff. Nevertheless, the government and HSE did support community facilities during COVID-19, not least with the establishment of HSE COVID-19 Response Teams to prevent, identify, and manage COVID-19 outbreaks across these services (public and private); direct invention of Defence Forces for cleaning and catering duties; and to redeploy staff, largely nurses and health care assistants, to support these community facilities [38]. These unprecedented measures also drew attention to the ever-growing privatisation of community care, with private nursing home staff, in 461 private and voluntary facilities (80% of all nursing homes), doubling during the study period from 18,000 in 2007 to 36,000 in 2019, and GPs growing by 10% from 2014 to 2021 [38–41].

In the immediate term, there were clear adverse effects on broader community health and social care during the COVID-19 period, with increased waiting lists for all allied health professionals—analysed in a recent paper examining healthcare activity in Ireland during the first 9 months of the pandemic [32]. While some specialties were able to curb the growing waiting lists by adopting telecare options, others were not. McGlacken-Byrne et al. specifically indicated significant decreases in the proportion of infants receiving a 10-month developmental screening within usual timeframes from a public health nurse as well as increased waiting lists to access care in the community, which were already high pre-COVID-19. Disruption to non-COVID-19 specific medical needs, particularly community-based services has been repeated worldwide, with 93% of 130 countries recently surveyed reporting disruption to mental health services [42].

### Reorganisation and governance

It is important to note that the data presented in this study, during the COVID-19 period, did not consider the redeployment of frontline staff. These changes were not captured in the national dataset, since people’s recorded place of employment did not change, instead their role and often place of work was temporarily changed. The ‘official statistics’ therefore potentially misrepresent the actual distribution of HSE staff. There were several examples of community-based redeployment (i.e., staff redeployed to community-based testing and vaccination centres, along with triaging of patients at primary care, often from a patient’s home via telecare), however the recorded place of employment remains largely weighted in favour of acute settings. This over-emphasis on hospitals contradicts the mammoth efforts to achieve integrated care delivery in community and primary care settings [26]. As Buchan et al. [35] recommends a rethinking of workforce governance is required as we move beyond the pandemic, in order to mobilise, train and deploy sufficient health and care workers with the necessary skills, whilst also making effective use of technology—the health system workforce must be “protected” by supporting and enabling staff to recover, rebuild and repurpose. To achieve this, key decisions must be channelled through (1) national/regional government policies (e.g., healthcare, education and employment); (2) legislation (e.g., working hours and prescribing); (3) regulation (e.g., professional councils defining roles and standards); and (4) the role and remit of employers and management (e.g., determining pay levels and working patterns) [35].

Furthermore, this analysis does not capture GPs (25% of practising doctors in Ireland [43], who act as gatekeepers for the vast majority of specialised testing and treatment within acute settings) or those in private hospitals and nursing homes who provided essential care during the pandemic. In reality, both public and private health providers worked collaboratively. While many of these changes posed unprecedented governance challenges within health systems in Europe and worldwide, for example legislative changes; negotiations with professional bodies; government approval for additional funding; and authority for allied health professionals to perform vaccinations to name but a few [35]. Learning from these rapid governance changes is required to strengthen workforce resilience into the future.

### Staff recovery and rebuilding

While acknowledging the achievements during the COVID-19 period, both in terms of strategic policy advancements and the apparent resilience of the health system to function under extreme and unchartered conditions, these changes came at a huge psychological, physical and social costs to the workforce. The COVID-19 pandemic has had a detrimental impact on staff health and well-being internationally, particularly for those working in long-term residential care facilities [44], on COVID-19 wards, in the ambulance service, emergency departments and ICU [45]. Despite this, the overall drop in non-COVID-19 absence-rates presented in this study implies workforce resilience in the short-term, with COVID-19 infections explaining the short-term spike in absences overall. Nevertheless, research conducted by the Irish Nurses and Midwives Organisation, indicated longer-term challenges with 90% of 1905 survey respondents indicating mental exhaustion. This startling figure also translated to 68% of respondents considering leaving the profession as a consequence of COVID-19 [46]. Nurses were identified at particular risk of burnout, in another international systematic review conducted during the early stages of the COVID-19 pandemic [47]. While some doctors reported an initial up-lift in physical well-being during the initial stages of the pandemics, sustained pressure also led to anxiety, emotional exhaustion, guilt, and isolation among this cohort [48]. These challenges come on foot of a prolonged period of austerity, with increasing concern about the cumulative effect on workforce absence, burnout and the potential of higher levels of turnover and early retirement [35]. Together, with a global shortage of health staff [49], this makes implementation of the 2021 resourcing plan very difficult, combating the potential for increasing attrition while attempting to find an additional 16,000 WTE by end of 2022 [24].

During a global pandemic, it may seem naive to suggest reducing workplace stressors or adjusting work shifts to prevent or reduce mental health issues and burnout [45]. This said, the health workforce is the central plank of providing all health and social care and they have continued to sustain extreme pressure throughout the pandemic. Pre-pandemic problems persist and some, for example waiting lists, are getting worse as a result. Growing demand and diminishing working conditions, further complicated by inadequate staffing levels to cover statutory leave, mean that staff are increasingly likely to suffer consequences of exhaustion and burnout [50]. It is imperative that the experiences, motivations and underlying mechanisms of staff resilience are captured and translated into policy to ensure a sustainable model of staff recruitment and retention is put in place that protects staff health and well-being, while also ensuring sufficient coverage.

### Limitations

While the HSE is the largest public sector employer in Ireland and main funder of the Irish health system, the national dataset does not contain information related to individuals who are not directly employed by the HSE, including agency staff; GPs (of which there are approximately 3300 contracted to provide services on behalf of the HSE in Ireland [51]); private hospitals; healthcare professionals practising privately; and the majority of care homes and residential settings for older people and those living with a disability (which are largely privately owned, charitable and/or religious organisations).

## Conclusion

Over the past 14 years, the Irish health system has experienced two major global shocks, with unprecedented change and challenges for the health workforce. The analysis presented in this paper clearly demonstrates the prioritisation of staff recruitment within acute services over the study period. While increasing capacity in acute sector is a policy priority, the data presented do not demonstrate a commitment to recruit the required level of staff to achieve government policy to shift care into primary and community care.

Some of the responses to COVID-19 were aligned with the health system reform agenda, namely a universal community-based approach to COVID-19 care, while also demonstrating the potential for collaboration and clearer governance between and within public and private health care providers. While the response to COVID-19 should be commended in terms of the agility of the system and the evident commitment from staff, the mismatch between policy and practice in recent years highlights the need to revisit workforce recruitment strategies to ensure the right skill-mix is available in the right (community) settings. Concerted action, infrastructure, and the permanent redistribution of personnel to ensure progressive and sustainable lessons are learned from recent shocks. Not only is direct action required to align government policy with practice, but it is also essential to protect and support those already in situ to ensure a well-resourced, appropriately located and resilient workforce into the future.

## Data Availability

The data that support the findings of this study are available from the National Health Service Executive Human Resources Directorate, but restrictions apply to the availability of these data, which were used under license for the current study, and so are not publicly available. Data are however available from the authors upon reasonable request and with permission of the Health Service Executive.

## Abbreviations

DoH: Department of Health
GPs: General practitioners
HSE: Health Service Executive
WTE: Whole-time equivalent/equivalence
